# Nanoparticle Contrast-enhanced T1-Mapping Enables Estimation of Placental Fractional Blood Volume in a Pregnant Mouse Model

**DOI:** 10.1038/s41598-019-55019-8

**Published:** 2019-12-10

**Authors:** Andrew A. Badachhape, Laxman Devkota, Igor V. Stupin, Poonam Sarkar, Mayank Srivastava, Eric A. Tanifum, Karin A. Fox, Chandrasekhar Yallampalli, Ananth V. Annapragada, Ketan B. Ghaghada

**Affiliations:** 1 Department of Radiology, Baylor College of Medicine, Houston, TX 77030 USA; 2 Department of Pediatrics-Oncology, Baylor College of Medicine, Houston, TX 77030 USA; 3 The Singleton Department of Pediatric Radiology, Texas Children’s Hospital, Houston, TX 77030 USA; 4 Department of Obstetrics and Gynecology, Texas Children’s Hospital, Houston, TX 77030 USA

**Keywords:** Molecular medicine, Preclinical research

## Abstract

Non-invasive methods for estimating placental fractional blood volume (FBV) are of great interest for characterization of vascular perfusion in placentae during pregnancy to identify placental insufficiency that may be indicative of local ischemia or fetal growth restriction (FGR). Nanoparticle contrast-enhanced magnetic resonance imaging (CE-MRI) may enable direct placental FBV estimation and may provide a reliable, 3D alternative to assess maternal-side placental perfusion. In this pre-clinical study, we investigated if placental FBV at 14, 16, and 18 days of gestation could be estimated through contrast-enhanced MRI using a long circulating blood-pool liposomal gadolinium contrast agent that does not penetrate the placental barrier. Placental FBV estimates of 0.47 ± 0.06 (E14.5), 0.50 ± 0.04 (E16.5), and 0.52 ± 0.04 (E18.5) were found through fitting pre-contrast and post-contrast T1 values in placental tissue using a variable flip angle method. MRI-derived placental FBV was validated against nanoparticle contrast-enhanced computed tomography (CE-CT) derived placental FBV, where signal is directly proportional to the concentration of iodine contrast agent. The results demonstrate successful estimation of the placental FBV, with values statistically indistinguishable from the CT derived values.

## Introduction

The placenta is a critical organ for nutrient transportation and oxygen exchange between fetal and maternal systems. This exchange is dependent on the rate of both uterine and umbilical blood flow, which in turn depends upon normal placental implantation and remodeling of maternal spiral arteries. Fetal growth potential is dependent on sufficient rate of exchange, which can be reduced due to placental disorders^1^. A reduced rate of transplacental exchange, often termed placental insufficiency, can impair fetal growth, leading to fetal growth restriction (FGR) as well as stillbirth^2^. Vascular perfusion is a determinant of transplacental transport and vascular hypo-perfusion is considered indicative of placental ischemia^3^. Placental fractional blood volume (FBV) is indicative of perfusion and may therefore identify both the potential for reduced transplacental transport and local ischemia. Non-invasive methods for the estimation of placental FBV are thus of great interest for characterizing placental perfusion.

Impedance of blood flow in the placenta is a critical factor in determining the rate of transplacental exchange and is often assessed indirectly through indices of placental flow in the umbilical artery. Indices such as the umbilical artery systolic-to-diastiolic ratio, resistance index, or pulsatility index may be measured through frequency-based color Doppler ultrasound^4–7^. This is used clinically only after FGR has been identified or there is evidence that placental insufficiency has already had clinical impact. Further, ultrasound is often used to monitor fetal well-being and avert stillbirth. Previous ultrasound studies have observed that the inability to detect intraplacental flow signal^8^ or reduced villous development^9^ may correlate with FGR, however variation in Doppler signal along with the difficulty in imaging intraplacental arteries can limit the effectiveness of frequency-based color Doppler of the placenta itself^10,11^.

Developments in 3D power ultrasound have enabled new methods for characterizing placental tissue and fetal organs. Common power ultrasound metrics used to assess 3D flow include vascularization index (VI), flow index (FI), and vascular-flow index (VFI)^7,10,12^. One proposed method for assessing placental vascularity is estimation of fractional moving blood volume (FMBV) through 3D power ultrasound, which is calculated as the ratio of Doppler power in the target organ (the placenta) to Doppler power in a large blood vessel set as a reference for 100% vascular amplitude^12–14^. Despite the prevalence and utility of ultrasound in assessing abnormalities throughout pregnancy, there is substantial variation in Doppler signal due to attenuation from intervening tissue, individual machine settings, and operator skill, which limit the clinical utility of 3D power ultrasound in estimating FMBV^13,15^. Estimates for FMBV using color Doppler can vary widely with mean estimates of 11.78% ± 9.3% (range: 0.012–44.16%)^13^.

Non-invasive, early detection of abnormally low placental FBV could help determine which pregnancies are truly at risk for poor clinical outcomes. Discrepancies in ultrasound estimates of placental FMBV demonstrate a need for a more definitive measure of placental perfusion^13,15^. Placental FBV measured using MRI and a blood-pool contrast agent should provide accurate estimates of perfusion by calculation of placental signal enhancement compared to that of a fully vascularized compartment. As this can be performed non-invasively, CE-MRI provides a unique window into understanding placental function even in early pregnancy and following the natural history to delivery. Absence of radiation and 3D imaging capabilities make CE-MRI well-suited for assessing placental FBV given the current limitations of ultrasound. Further, consistent placental FBV estimated from CE-MRI may be used to validate and improve ultrasound methods for calculating FMBV.

MRI is relatively more expensive to perform, requires technical expertise and takes up more space than ultrasound. Ultrasound remains the primary imaging method for assessing the health of pregnancy, however MRI is often utilized as well, particularly in tertiary/quaternary centers for cases in which ultrasound findings are unclear, such as to assess fetal anomalies of the brain, fetal lung volumes in cases of congenital diaphragmatic hernia, or for invasive placenta^16,17^. We anticipate that use of CE-MRI to detect decreased placental FBV prior to the onset of fetal growth restriction may help guide clinicians in counseling patients and in managing high-risk pregnancies.

Though gadolinium-enhanced MRI may prove useful for assessing abnormalities related to placental perfusion, its translational potential is critically linked to concerns about fetal gadolinium exposure. Clinical studies have demonstrated that gadolinium contrast agents have a very low incidence of side effects in the mother, however fetal risks are less certain. For instance, one study considering the clinical use of gadolinium-enhanced MRI for fetal imaging found an overall low risk to both mother and fetus^18^. Another large study, however, found that Gd exposure in the second and third trimesters resulted in a host of conditions ranging from rheumatological and inflammatory conditions to stillbirth^19^. Due to these concerns, the risks and benefits of Gd use are assessed on a per-patient basis before administration^16,18^.

We have previously demonstrated that a long circulating liposomal nanoparticle-based blood-pool Gd contrast agent (liposomal-Gd) does not penetrate the placental barrier in rodent models, thereby sparing the fetus from placental exposure^20,21^. Furthermore, due to its high T1 relaxivity and long blood half-life, it enables high-resolution visualization of placenta and its margins including the retroplacental clear space^22^. In this pre-clinical study, we investigated if the properties of this liposomal-Gd contrast agent would yield accurate estimation of placental FBV in a pregnant mouse model.

## Results

**Figure 1:**
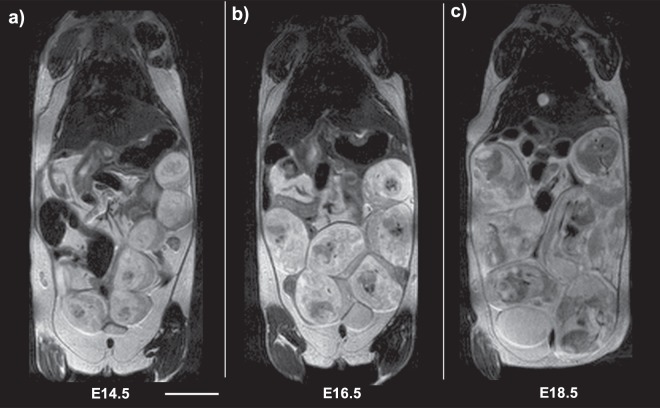
T2-weighted anatomical coronal images of pregnant animals at (**a**) E14.5, (**b**) E16.5, and (**c**) E18.5 days of gestation demonstrate fetal growth and feto-placental unit (FPU) development. Scale bar represents 1 cm.

Pregnant female C57BL/6 J mice were imaged on days 14, 16, and 18 of pregnancy (referred to hereafter as E14.5, E16.5, and E18.5). Since each mouse bears multiple feto-placental units (FPU), we were able to analyze 24 FPU at E14.5, 20 FPU at E16.5, and 23 FPU at E18.5. T2-weighted fast spin echo (T2w-FSE) anatomical images of mouse abdomen enabled identification of placenta and fetal compartment at E14.5, E16.5, and E18.5 (Fig. 1). As gestation progressed, both the fetal compartment and placenta expanded in volume.

**Figure 2:**
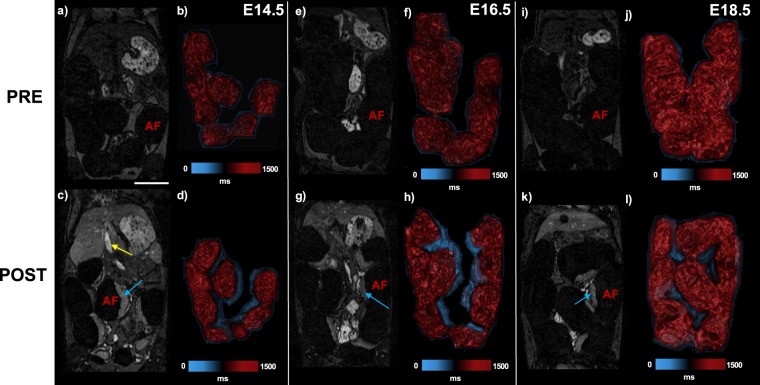
Nanoparticle contrast-enhanced MRI (CE-MRI) enables T1 mapping of the placenta. Pre-contrast (top row) and post-contrast (bottom row) T1-weighted gradient recalled echo (T1w-GRE) coronal images at E14.5 (**a,c**), E16.5, (**e,g**), and E18.5 (**i,k**) show signal enhancement in the placenta (blue arrow) and IVC (yellow arrow) after administration of liposomal-Gd. No signal is seen in the amniotic fluid (AF) compartment of the feto-placental unit. Volume renderings of corresponding feto-placental units for pre-contrast and post-contrast scans are shown for E14.5 (**b,d**), E16.5 (**f,h**), E18.5 (**j,l**). Color bar indicates T1 relaxation times in milliseconds(ms). Scale bar represents 1 cm.

**Figure 3:**
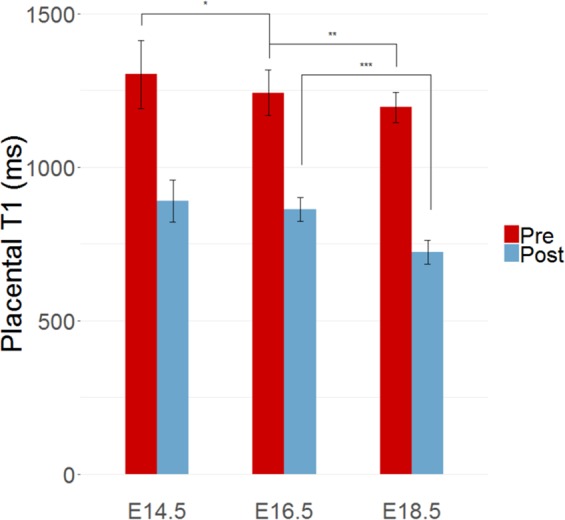
Placental T1 relaxation time estimates decrease following injection of liposomal-gadolinium contrast agent. Post-contrast T1 values are significantly lower than pre-contrast T1 values (p < 0.0005). Pre-contrast T1 values decreased as gestation progressed from E14.5 to E18.5. Post-contrast T1 values in the placenta were significantly lower at E18.5 than at E16.5. T1 relaxation times are reported in milliseconds (ms). Significance as determined by the Wilcoxon rank sum test are shown for p < 0.05 (one asterisk), p < 0.005 (two asterisks), and p < 0.0005 (three asterisks).

T1-weighted gradient recalled echo (T1w-GRE) images (α = 70°) demonstrated uniform and stable signal enhancement in placenta and inferior vena cava (IVC) in post-contrast MRI scans at E14.5, E16.5, and E18.5 (Fig. 2). T1-mapping using the variable flip angle method showed large decreases in post-contrast T1 relaxation times in placenta (~30–40%) and IVC (~30–55%), but not in the fetal compartment (Fig. 2). Post-contrast T1 relaxation times in the IVC and placenta were decreased by a similar amount at E14.5 (~30%), however the IVC demonstrated larger decreases in T1 relaxation time at E16.5 and E18.5 compared to the placenta (Table 1). Pre-contrast T1 values in the placenta were significantly different from each other at each of the three gestational time points. Post-contrast T1 values in the placenta were significantly lower at E18.5 than at E14.5 and E16.5 (Fig. 3).

**Table 1:** Pre-contrast and post-contrast estimates of T1 relaxation times in the placenta and inferior vena cava (IVC) at each of the three timepoints.

	Placenta T1 (ms)		IVC T1 (ms)	
	Pre	Post	Pre	Post
E14.5	1300 ± 110	890 ± 70	1020 ± 60	690 ± 60
E16.5	1240 ± 70	860 ± 40	1150 ± 40	600 ± 40
E18.5	1200 ± 50	720 ± 40	1060 ± 30	480 ± 30

T1 values are reported as mean and standard deviation in milliseconds (ms).

**Figure 4:**
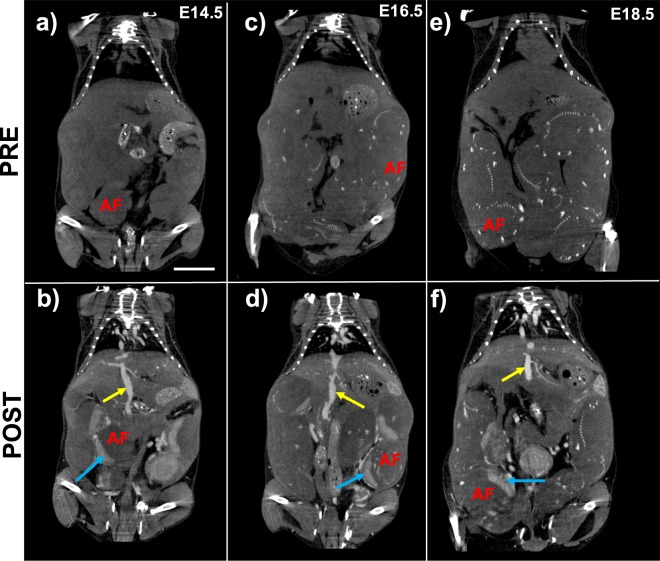
Nanoparticle contrast-enhanced computed tomography (CE-CT) demonstrates uniform vascular enhancement and enables validation of MRI-derived placental fractional blood volume (FBV). Comparison of pre-contrast and post-contrast coronal images for (**a,b**) E14.5, (**c,d**) E16.5, and (**e,f**) E18.5 show signal enhancement in the IVC (yellow arrow) and placenta (blue arrow). Fetal skeletal features within the amniotic fluid (AF) compartment are visible in both pre-contrast and post-contrast images and become more pronounced at later stages of gestation. Scale bar represents 1 cm.

Contrast-enhanced CT (CE-CT) images demonstrated in Fig. 4 also showed uniform and stable enhancement in placenta and IVC in post-contrast images (Fig. 4). Fetal skeletal anatomy became more apparent at E16.5 and E18.5.

**Figure 5:**
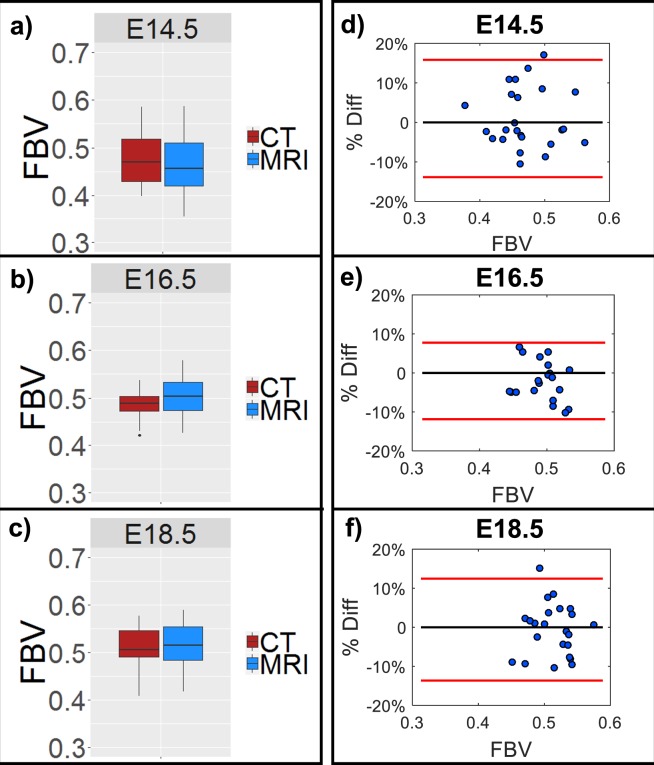
Placental fractional blood volume (FBV) estimated from nanoparticle contrast-enhanced MRI (CE-MRI) shows close agreement with values estimated by nanoparticle contrast-enhanced computed tomography (CE-CT). Box-and-whisker plots shown for (**a**) E14.5, (**b**) E16.5, and (**c**) E18.5 demonstrate mean placental FBV (horizontal lines) and standard deviation (vertical lines) along with any outliers (·) for CT (red) and MRI (blue). Bland-Altman plots showing the percent difference between MRI and CT estimates of FBV for each individual placenta are also shown for (**d**) E14.5, (**e**) E16.5, and (**f**) E18.5. Percent difference measurements for each individual FPU are represented (blue dots) along with the 95% confidence interval (red lines).

Placental FBV estimated from CE-MRI showed close agreement with values estimated by CE-CT at each of the three time points (Fig. 5). Bland-Altman analysis of MRI-derived and CT-derived FBV on a per FPU basis demonstrated that 97% of all estimates fell within the confidence interval (Fig. 5). MRI-derived placental FBV: [0.47 ± 0.06 at E14.5; 0.5 ± 0.04 at E16.5; 0.52 ± 0.04 at E18.5] showed strong agreement with CT-derived placental FBV: [0.48± 0.06 at E14.5; 0.48 ± 0.03 at E16.5; 0.51 ± 0.04 at E18.5]. Absolute mean difference between individual MRI-derived and CT-derived FBV estimates were: 6.2% (E14.5), 4.4% (E16.5), and 5.3% (E18.5). Both MRI and CT estimates of placental FBV found a statistically significant increase in FBV estimates between E14.5 and E18.5 (see Supplementary Table S1 and Supplementary Table S2 online).

## Discussion

Contrast-enhanced T1-mapping using a variable flip angle method with a liposomal-Gd contrast agent enabled accurate estimation of placental fractional blood volume in late gestation of a murine model of pregnancy. As shown in previous studies^21,22^, liposomal-Gd enables characterization of feto-placental growth and vascular development during gestation. The high T1 relaxivity of the liposomal-Gd contrast agent resulted in shortened T1 relaxation times in placental tissue and large blood vessels such as the IVC.

Mean placental FBV estimates derived from contrast-enhanced T1-mapping showed close agreement with paired estimates from contrast-enhanced CT. At all three gestational ages, 97% of MRI-derived estimates of placental FBV fell within the 95% confidence interval determined by Bland-Altman analysis, thus indicating that CE-MRI and CE-CT measures of placental FBV are interchangeable. No statistical significance was found between CE-MRI and CE-CT estimates of placental FBV at E14.5 (p = 0.66), E16.5 (p = 0.11), or E18.5 (p = 0.78). Placental FBV estimates from both modalities show a slight but statistically significant increase between E14.5 and E18.5.

The observed increase in placental FBV estimates between E14.5 and E18.5 support previous findings regarding increased vascular development in the mouse placenta as gestation progresses^23,24^. Clinical ultrasound studies have also demonstrated increased vasculature development through gestation^25,26^, however there is significant variation in the estimates of vascular indices throughout these studies. These results demonstrate the potential diagnostic capabilities of liposomal-Gd for identifying placental vasculature disorders.

Ultrasound and conventional MRI are, respectively, primary and secondary options for assessing the health of pregnancy, however neither can provide accurate estimates of placental FBV. CE-MRI holds great promise as a tool for estimating placental FBV, particularly in high-risk pregnancies or cases where ultrasound findings are unclear. Additionally, liposomal-Gd enhanced MRI may serve as a measurement standard for improving ultrasound assessment of placental perfusion. Future pre-clinical studies with this agent will seek to characterize earlier gestational time frames and compare CE-MRI estimates of placental FBV with ultrasound measures of FMBV.

Our study had several limitations. Fetal motion is a large source of motion artifact when performing MRI of pregnant animals and cannot be gated for in our scan protocol. Additionally, maternal respiratory motion was not gated for in our acquisition. We did not perform gating as we have found that a better artifact management strategy for coping with combined respiratory and fetal motion is to assess individual acquisitions and repeat the scans if large motion artifact is present^21,22^. GRE scans were reacquired in three cases in this study: two subjects in the E16.5 cohort and one subject in the E18.5 cohort. Additionally, since our contrast agent does not cross into fetal circulation, our placental FBV estimates can only asses the maternal side of the placental volume. Our use of the IVC as a reference for pure blood-pool signal may lead to some discrepancies in estimates due to the relative difference of the IVC diameter (measured as ~2 mm) compared with our GRE voxel size (0.5 mm isotropic voxels). As with previous studies performed by our group^22^, only three animals were scanned with both MRI and CT at each of the three time points. The dams bear 6–10 feto-placental units (FPU), which allowed us to test 20 to 24 FPU on MRI and CT at each of the three time points. Since placental FBV was estimated on a per-FPU basis, utilizing three animals enabled us to minimize the number of dams that were sacrificed for this study without compromising statistical analysis of data.

T1-mapping using a variable flip angle method with a liposomal-Gd blood-pool contrast agent for MRI enabled accurate determination of placental fractional blood volume as compared with contrast-enhanced CT, where signal intensity is proportional to iodine concentration. Implementing a variable flip angle method at a clinically relevant field strength of 1 T further demonstrates the potential viability of this approach in characterizing placental vascular perfusion during pregnancy. Contrast-enhanced MRI using liposomal-Gd may serve as an alternate 3D-based imaging technique for measurement of placental FBV and determining potential placental vascular abnormalities.

## Materials and Methods

All animal studies were performed under a protocol approved by the Institutional Animal Care and Use Committee of the Baylor College of Medicine. All studies were in accordance with NC3RS-ARRIVE guidelines.

### Animal model

Pregnant female C57BL/6J mice (8–12 weeks old; ~20–30 g body weight before pregnancy) were imaged on days 14, 16, and 18 of pregnancy (E14.5, E16.5, and E18.5). Since C57BL/6J mice will deliver around approximately 20 to 21 days, our experimental group covers late gestation and a period roughly analogous to the third trimester in humans. Separate cohorts of pregnant animals were imaged at each of the three gestational ages (n = 3 pregnant animals per cohort per time point) to eliminate effects of residual contrast agent beyond the first imaging time point. The first day of gestation, designated as E0.5, was when a vaginal copulation plug was detected. Since each dam developed between 6 and 10 feto-placental units (FPU), we were able to analyze 24 FPU at E14.5, 20 FPU at E16.5, and 23 FPU at E18.5.

### Liposomal-Gd contrast agent

Liposomal-Gd contrast agent was prepared per procedures described previously^21^. Briefly, hydrogenated soy phosphatidylcholine (HSPC), 1,2-distearoyl-sn-glycero-3-phosphoethanolamine 1,4,7,10-Tetraazacyclododecane-1,4,7,10-tetraacetic acid Gadolinium (III) (DSPE-DOTA-Gd), cholesterol, and 1,2-distearoyl-sn-glycero-3-phosphoethanolamine-N-[methoxy(- poly(ethylene glycol))-2000] (mPEG2000-DSPE) were dissolved in t-butanol at a molar ratio 31.5:25:40:3.5. The lipid solution was hydrated with 150 mM NaCl/10 mM histidine to achieve a lipid concentration of 75 mM. The solution was stirred for 30 minutes at 60 °C and then sequentially extruded on a Lipex Thermoline extruder through 200 nm polycarbonate track-etch membranes. The resulting liposomal suspension was dialyzed against 150 mM NaCl/10 mM histidine. The mean liposome size in the final formulation, determined by dynamic light scattering (DLS), was 122 nm with a poly-dispersity index of less than 0.15. The gadolinium and phospholipid (equivalent phosphorus) concentrations in the liposomal formulation, quantified using inductively coupled plasma optical emission spectroscopy (ICP-OES), were 13 mM and 33 mM, respectively. For *in vivo* studies, liposomal-Gd was administered intravenously via the tail vein at a dose of 0.1 mmol Gd/kg body weight.

### Magnetic resonance imaging (MRI)

Imaging was performed on a 1 T permanent MRI scanner (M2 system, Aspect Imaging, Shoham, Israel), incorporating a 35 mm transmit-receive RF volume coil. Animals were sedated using 3% isoflurane, placed on the MRI animal bed, and then maintained at 1–2% isoflurane delivered using a nose cone setup. Respiration rate was monitored by a pneumatically controlled pressure pad placed underneath the abdominal region of the animals.

All animals were scanned before and after liposomal-Gd contrast administration. Anatomical T2-weighted (T2w) scans were acquired using a fast spin echo (FSE) sequence. Scan parameters for T2w-FSE scans were: echo time (TE) = 80 ms, repetition time (TR) = 6816 ms, slice thickness = 0.8 mm, field of view = 80 mm × 80 mm, number of slices = 33, matrix = 256 × 250, acquisition plane = coronal; in-plane resolution = 312.5 × 320 µm^2^, number of excitations = 2, echo train length = 2, scan time ~ 6 min.

T1-mapping was calculated using a variable flip angle study wherein pre-contrast and post-contrast images were acquired using a T1-weighted 3D gradient-recalled echo sequence (T1w-GRE). Scan parameters for the T1w-GRE sequence were: echo time (TE) = 3.5 ms, repetition time (TR) = 20 ms, flip angle (*α*) = [8°, 15°, 25°, 35°, 45°], slice thickness = 0.5 mm, field of view = 54 mm × 54 mm, number of slices = 80–100, matrix = 180 × 180, acquisition plane = coronal; in-plane resolution = 500 × 500 µm^2^, scan time ~5 minutes. Receiver gain was held at the same level between flip angle changes. GRE scans at each flip angle were assessed for motion immediately after acquisition and all five scans were reacquired if large motion artifact was present. GRE scans were repeated in two subjects in the E16.5 cohort and one subject in the E18.5 cohort due to motion artifact. A high resolution T1w-GRE sequence (0.3 mm isotropic voxels, α = 70°) with two external averages was also acquired to better visualize placental margins. Post-contrast images were acquired following intravenous administration of liposomal-Gd (0.1 mmol Gd/kg).

The signal equation for a gradient-recalled echo sequence (Eq. 1) describes the relationship between T1 relaxation time, T2 relaxation time, and apparent signal for each voxel at a given flip angle *α*_*i*_^27^.

1 $${S}_{{\alpha }_{i}}=\frac{{M}_{0}(1-{e}^{-\frac{TR}{T1}}){e}^{-\frac{TE}{T2}}}{1-cos({\alpha }_{i})\cdot {e}^{-\frac{TR}{T1}}\,}\,\sin ({\alpha }_{i})$$

Here, *M*_0_ describes total proton density and instrument related parameters and is assumed to be the same between measurements at different flip angles. In T1-weighted imaging, where TE is much shorter than T2 relaxation time, the term ${e}^{-\frac{TE}{T2}} \sim 1$:

2 $${S}_{{\alpha }_{i}}=\frac{{M}_{0}(1-{e}^{-\frac{TR}{T1}})}{1-cos({\alpha }_{i})\cdot {e}^{-\frac{TR}{T1}}\,}\,\sin ({\alpha }_{i})$$

This expression can then be further simplified:

3 $$\frac{{S}_{{\alpha }_{i}}}{\sin ({\alpha }_{i})}=\frac{{S}_{{\alpha }_{i}}}{\tan ({\alpha }_{i})}\cdot {e}^{-\frac{TR}{T1}}+{M}_{0}(1-{e}^{-\frac{TR}{T1}})$$

Placental T1 relaxation times (*T*1_*p*_) were calculated by fitting mean signal intensity (*Sα*_*i*_) in a region of interest (ROI) that encompassed the entire 3D placental tissue volume against the corresponding flip angle using Eq. 3. Analysis was performed for both pre-contrast and post-contrast images. Placental tissue ROI in pre-contrast T1w-GRE images were found through comparison with T2w-FSE images. Mean signal intensities in ROI sampled in the inferior vena cava (IVC) were used to calculate the T1 of blood (*T*1_*IVC*_). T1 relaxation rate (*R*1=1/*T*1) was then calculated for the placenta (*R*1_*P*_) and IVC (*R*1_*IVC*_). MRI-derived placental fractional blood volume (*FBV*_*MRI*_) was calculated as the ratio of increase in relaxation rate between placenta and IVC according to Eq. 4.

4 $$FB{V}_{MRI}=\varDelta \,R{1}_{P}/\varDelta \,R{1}_{IVC}$$

Three pregnant animals were imaged with MRI at E14.5, E16.5, and E18.5, giving a total subject pool of nine mice. Animals were scanned with computed tomography immediately after completion of MRI studies. Mean placental FBV estimates were calculated from the total pools of 24 FPU at E14.5, 20 FPU at E16.5, and 23 FPU at E18.5.

### Computed tomography (CT) imaging

Contrast-enhanced CT (CE-CT) imaging was used for validation of MRI-derived placental FBV. CE-CT was performed using a long circulating blood-pool contrast agent similar to methods described previously^22^. Briefly, animals were scanned on a small animal micro-CT system (Siemens Inveon) approximately one hour after MRI studies had been completed. Animals were sedated with 3% isoflurane, positioned on the CT animal bed, and then maintained at 1–2% isoflurane delivered via nose cone. A pneumatically controlled pressure pad was placed underneath the animal’s abdominal region to monitor respiration rate during the CT imaging session.

The scan parameters for the CT image acquisition were: 50 kVp, 0.5 mA, 850 ms X-ray exposure, 540 projections, 35 µm isotropic spatial resolution, scan time ~ 10 minutes. The acquired X-ray projection images were reconstructed into 3D datasets using a filtered back-projection reconstruction algorithm. All datasets were Hounsfield Unit (HU) calibrated for image analysis. CE-CT was performed after intravenous administration of a liposomal-iodinated blood-pool contrast agent (1.65 mg I/g body weight)^28–30^. Upon completion of imaging studies, animals were euthanized by CO_2_ inhalation.

CT-derived placental FBV (*FBV*_*CT*_) was calculated as the ratio of signal enhancement in placenta to IVC between pre-contrast and post-contrast scans (Eq. 5).

5 $$FB{V}_{CT}=\varDelta \,H{U}_{P}/\varDelta \,H{U}_{IVC}$$

Here, *HU*_*P*_ describes the mean CT signal (expressed in HU) in an ROI encompassing the entire 3D placental volume while *HU*_*IVC*_ similarly describes the mean CT signal in the IVC.

### Quantitative analysis

The Wilcoxon rank sum test was used for statistical analysis of placental T1 relaxation times and placental FBV between E14.5, E16.5, and E18.5. Bland-Altman analysis^31^ was performed to assess differences in MRI-derived estimates of placental FBV with the standard provided by CT data. Plots at each of the three gestational time points were generated with the 95% confidence interval calculated as ±1.96 standard deviation of the difference in estimated placental FBV from both modalities.

## Supplementary information

Supplementary Material - Tables

## Data Availability

Data supporting the findings of this study are available from the corresponding author on request.
