# Supplementary material for: Nanoparticle Contrast-enhanced T1-Mapping Enables Estimation of Placental Fractional Blood Volume in a Pregnant Mouse Model
Source: Sci Rep. 2019 Dec 10;9:18707. doi: 10.1038/s41598-019-55019-8 (PMC6904754; doi:10.1038/s41598-019-55019-8)
Supplement: Supplementary file 1 — Supplementary Material - Tables [file 41598_2019_55019_MOESM1_ESM.pdf]

Supplementary Information

Nanoparticle Contrast-enhanced T1-Mapping Enables Estimation of Placental Fractional Blood Volume in a Pregnant Mouse Model

Authors and affiliations:

Andrew A. Badachhape, Ph.D., Department of Radiology, Baylor College of Medicine, Houston, TX 77030, USA, badachha@bcm.edu

Laxman Devkota, Ph.D., Department of Pediatrics-Oncology, Baylor College of Medicine, Houston, TX 77030, USA, laxman.devkota@bcm.edu

Igor V. Stupin, Ph.D., The Singleton Department of Pediatric Radiology, Texas Children's Hospital, Houston, TX 77030, USA, ivstupin@texaschildrens.org

Poonam Sarkar, Ph.D., Department of Pediatrics-Oncology, Baylor College of Medicine, Houston, TX 77030, USA, poonam.sarkar@bcm.edu

Mayank Srivastava, Ph.D., The Singleton Department of Pediatric Radiology, Texas Children's Hospital, Houston, TX 77030, USA, mxsrivas@texaschildrens.org

Eric A. Tanifum, Ph.D., The Singleton Department of Pediatric Radiology, Texas Children's Hospital, Houston, TX 77030, USA, eatanifu@texaschildrens.org

Karin A. Fox, M.D, Department of Obstetrics and Gynecology, Texas Children's Hospital, Houston, TX 77030, USA, kafox@bcm.edu

Chandrasekhar Yallampalli, D.V.M., Ph.D., Department of Obstetrics and Gynecology, Texas Children's Hospital, Houston, TX 77030, USA, chandrasekhar.yallampalli@bcm.edu

Ananth V. Annapragada, Ph.D., The Singleton Department of Pediatric Radiology, Texas Children's Hospital, Houston, TX 77030, USA, avannapr@texaschildrens.org

[*]Ketan B. Ghaghada, Ph.D., The Singleton Department of Pediatric Radiology, Texas Children's Hospital, Houston, TX 77030, USA, kbghagha@texaschildrens.org

[*]**Corresponding Author:**

Ketan B. Ghaghada, Ph.D.
The Singleton Department of Pediatric Radiology, Texas Children's Hospital
1102 Bates Street, Suite 850
Houston, TX 77030
Email: kbghagha@texaschildrens.org
Phone: 832-824-0865

Supplementary Information

	FBV p-values (CE-MRI)		
	**E14.5**	**E16.5**	**E18.5**
**E14.5**	-	0.022	0.002
**E16.5**	0.022	-	0.43
**E18.5**	0.002	0.43	-

Supplementary Table S1: Wilcoxon rank sum test p-values comparing mean placental fractional blood volume (FBV) estimates from contrast-enhanced MRI (CE-MRI) at each gestational timepoint. CE-MRI derived estimates of FBV are significantly different at E16.5 and E18.5 relative to E14.5.

	FBV p-values (CE-CT)		
	**E14.5**	**E16.5**	**E18.5**
**E14.5**	-	0.532	0.028
**E16.5**	0.532	-	0.018
**E18.5**	0.028	0.018	-

Supplementary Table S2: Wilcoxon rank sum test p-values comparing mean placental fractional blood volume (FBV) estimates from contrast-enhanced computed tomography (CE-CT) at each gestational timepoint. CE-CT derived estimates of FBV are significantly different at E18.5 relative to E16.5 and E14.5.
